# Genetically supported causality between gut microbiota, gut metabolites and low back pain: a two-sample Mendelian randomization study

**DOI:** 10.3389/fmicb.2023.1157451

**Published:** 2023-04-14

**Authors:** Mengchan Su, Yidan Tang, Weishuang Kong, Shuangyi Zhang, Tao Zhu

**Affiliations:** ^1^Department of Anesthesiology, West China Hospital, Sichuan University, Chengdu, China; ^2^Laboratory of Anesthesia and Critical Care Medicine, National-Local Joint Engineering Research Centre of Translational Medicine of Anesthesiology, West China Hospital, Sichuan University, Chengdu, China; ^3^Department of Surgery, Xuanwei Hospital of Traditional Chinese Medicine, Xuanwei, China

**Keywords:** Mendelian, gut microbiota, gut microbial metabolites, low back pain, sciatica, causality

## Abstract

**Background:**

Previous studies have implicated a vital association between gut microbiota/gut microbial metabolites and low back pain (LBP), but their causal relationship is still unclear. Therefore, we aim to comprehensively investigate their causal relationship and identify the effect of gut microbiota/gut microbial metabolites on risk of LBP using a two-sample Mendelian randomization (MR) study.

**Methods:**

Summary data from genome-wide association studies (GWAS) of gut microbiota (18,340 participants), gut microbial metabolites (2,076 participants) and LBP (FinnGen biobank) were separately obtained. The inverse variance-weighted (IVW) method was used as the main MR analysis. Mendelian randomization pleiotropy residual sum and outlier (MR-PRESSO) and MR-Egger regression were conducted to evaluate the horizontal pleiotropy and to eliminate outlier single-nucleotide polymorphisms (SNPs). Cochran’s *Q*-test was applied for heterogeneity detection. Besides, leave-one-out analysis was conducted to determine whether the causal association signals were driven by any single SNP. Finally, a reverse MR was performed to evaluate the possibility of reverse causation.

**Results:**

We discovered that 20 gut microbial taxa and 2 gut microbial metabolites were causally related to LBP (*p* < 0.05). Among them, the lower level of family *Ruminococcaceae* (OR: 0.771, 95% CI: 0.652–0.913, FDR-corrected *p* = 0.045) and *Lactobacillaceae* (OR: 0.875, 95% CI: 0.801–0.955, FDR-corrected *p* = 0.045) retained a strong causal relationship with higher risk of LBP after the Benjamini–Hochberg Corrected test. The Cochrane’s *Q* test revealed no Heterogeneity (*p* > 0.05). Besides, MR-Egger and MR-PRESSO tests showed no significant horizontal pleiotropy (*p* > 0.05). Furthermore, leave-one-out analysis confirmed the robustness of MR results. After adding BMI to the multivariate MR analysis, the 17 gut microbial taxa exposure-outcome effect were significantly attenuated and tended to be null.

**Conclusion:**

Our findings confirm the the potential causal effect of specific gut microbiota and gut microbial metabolites on LBP, which offers new insights into the gut microbiota-mediated mechanism of LBP and provides the theoretical basis for further explorations of targeted prevention strategies.

## 1. Introduction

Low back pain (LBP) is a symptom that refers to the pain and discomfort below the costal edge, above the buttock creases, and between the axillary midline, with or without leg pain (Koes et al., 2006). As the leading cause of years lived with disability worldwide, LBP is one of the most prevalent diseases with adverse societal impact (Chen et al., 2022). According to a systematic review including 165 studies from 54 countries, the point prevalence of LBP is 11.9 ± 2.0% (Hoy et al., 2012). Although LBP is usually self-limited, it’s estimated that 5–10% of people with LBP will develop chronic condition, which could result in higher socioeconomic burden and less measurable expenses such as problems doing household duties, caregiving, depression, and anxiety (Meucci et al., 2015). A growing number of medical practice guidelines have recommended many treatments to reduce the pain and its consequence, yet the management of LBP remains challenging (Bunzli et al., 2013; Waterschoot et al., 2014; Wertli et al., 2014). Given the high prevalence and heavy burden of LBP globally, there is an urgent need to identify potential causal risk factors for LBP.

The etiologies of LBP are multifactorial, including biological, psychological, and social factors (Knezevic et al., 2021). With 13 trillion bacterial cells, the human gut plays an important role in modulating host metabolites, vitamin production, colonization resistance, and immunological homeostasis. A growing body of evidence suggests that gut microbiota dysbiosis is associated with metabolic, immune, neurological and musculoskeletal disorders (Moon et al., 2018; Strandwitz, 2018; Boer et al., 2019). Recent studies suggest that gut microbiome may also be associated with pain condition including visceral pain, nociplastic pain, complex regional pain syndrome and headaches (Minerbi and Shen, 2022). A cohort study reported that patients with back pain showed a higher abundance of *Adlercreutzia*, *Roseburia*, and *Uncl. Christensenellacae* than controls in overweight and obese indic xviduals (Dekker Nitert et al., 2020). Additionally, it’s reported that the composition of the gut microbiota has been associated with pain conditions partly through altered concentration of gut microbial metabolites, highlighting the potential mechanisms involving the levels of circulating metabolites (Lührs et al., 2002; Shao et al., 2015; Patterson et al., 2019; Blaak et al., 2020). The dysregulation of gut microbial metabolites is potentially connected to pain (Li J. S. et al., 2022). However, the causal effect of gut microbiota and gut microbial metabolites on the risk of LBP has yet to be established because of potential biases.

Mendelian randomization (MR) is an efficient method for causality inference, utilizing genetic variants as instrumental variables (IVs) to research the causal effect of exposure on outcome (Katan, 1986; Smith and Ebrahim, 2003). This work selected gut microbiota and gut microbial metabolites as exposure and LBP as an outcome for MR analysis to explore the potential causal relationship, aiming to provide a theoretical basis for further research into the complex mechanisms and risk factors of LBP.

## 2. Materials and methods

### 2.1. Ethics approval statement

The summary-level data used in this study are available for download. Each GWAS involved in this study was ethically approved by the respective institutions.

### 2.2. Study design

**Figure 1:**
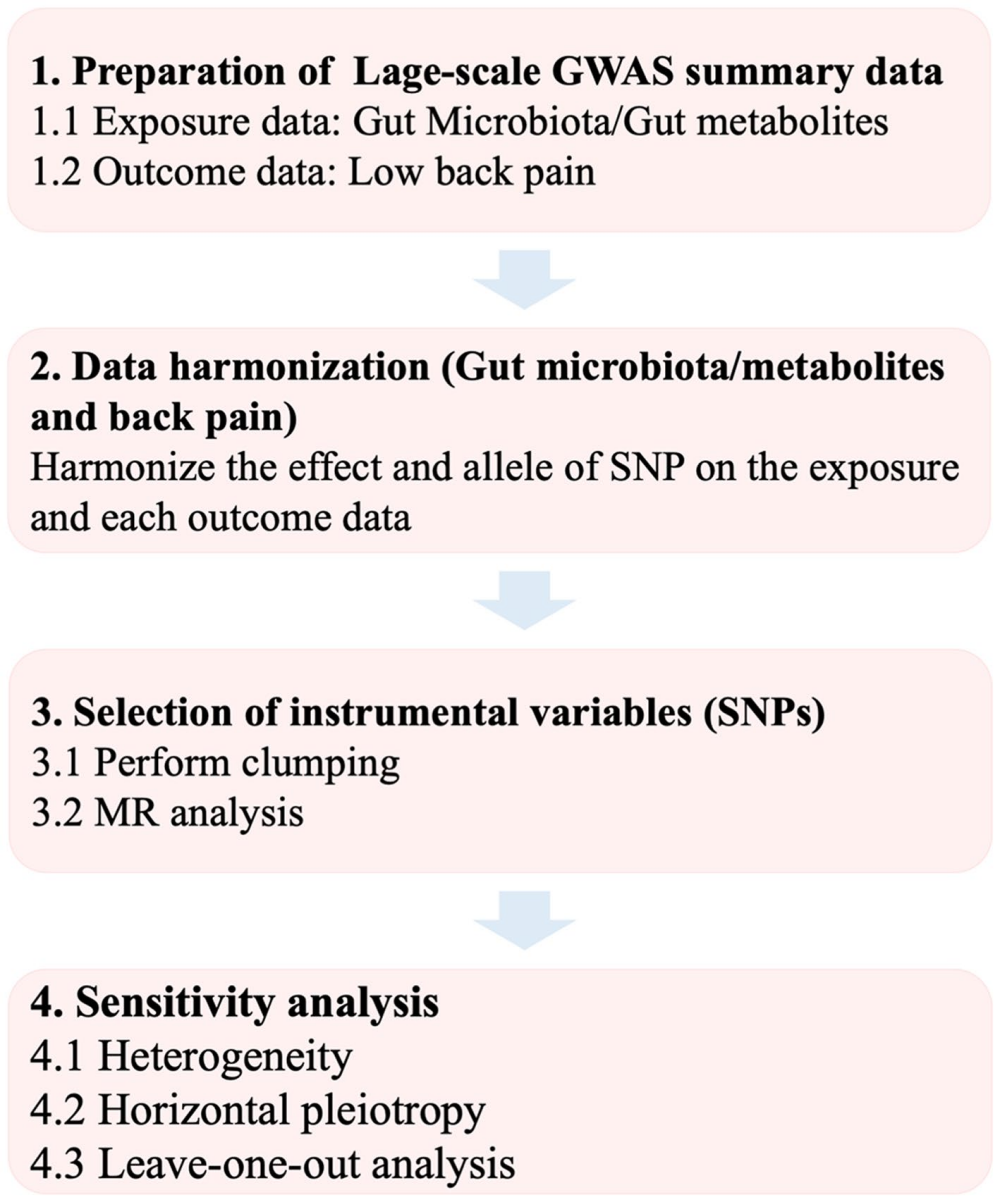
Flowchart of the study. GWAS, genome-wide association study; SNP, single-nucleotide poly-morphism; MR, Mendelian randomization.

Gut microbiota and gut microbial metabolites were selected as the exposure while the LBP served as the outcome. All statistics involved in the analysis were derived from publicly available genome-wide association studies (GWAS). Single-nucleotide polymorphisms (SNPs) associated with gut microbial taxa and gut microbial metabolites were extracted as IVs. Based on the summary-level data from GWAS of gut microbiota, gut microbial metabolites and LBP, we conducted a two-sample MR analysis. The flowchart of the study is shown in Figure 1.

### 2.3. Exposure data of gut microbiota and gut microbial metabolites

Summary statistics for gut microbiota were obtained from a large-scale GWAS study conducted by the MiBioGen consortium, which coordinated 16S rRNA gene sequencing profiles from 18,340 individuals (24 cohorts) (Kurilshikov et al., 2021). In total, 211 taxa (131 genera, 35 families, 20 orders, 16 classes, and 9 phyla) were included in the microbiome quantitative trait loci mapping analysis. Moreover, summary data for gut microbial metabolites were drawn from a GWAS study of the human metabolome, which was conducted among 2,076 participants (Rhee et al., 2013). Given the significance of microbiota-derived metabolites in microbiota-host interaction in nervous system and pain behavior, we chose key metabolites with available GWAS, including propionic acid, β-hydroxybutyric acid (BHB), serotonin, γ-aminobutyric acid (GABA), trimethylamine N-oxide (TMAO), betaine, choline, and carnitine. According to prior research, these gut microbial metabolites play critical roles in maintaining healthy nervous system, if dysregulated, are potentially causally connected to pain (Yang and Chiu, 2017; Guo et al., 2019; Li J. S. et al., 2022).

### 2.4. Outcome data of low back pain

The GWAS summary statistics of genetic associations for LBP were extracted from the largest GWAS meta-analysis, the FinnGen Biobank.^1^ After adjusting for age, sex, genetic relatedness, genotyping batch, and principal components, 13,178 LBP cases and 164,682 controls were used for analysis. In addition, considering that radicular pain or radiculopathy (previously called sciatica) sometimes present as LBP, 19,509 lower back pain or/and sciatica cases and 199,283 controls were also included for analysis (Maher et al., 2017; Table 1).

**Table 1:** Details of GWAS studies.

Phenotypes	Consortium	Population	Sample size (Case/Control)
Low back pain	FinnGen biobank	Europeans	13,178/164,682
Lower back pain or/and sciatica	FinnGen biobank	Europeans	19,509/199,283
Gut microbiota	MiBioGen	Europeans	18,340
Gut microbial metabolites	FHS Offspring Cohort	Europeans	2,076

FHS, Framingham Heart Study.

### 2.5. Genetic instruments selection and harmonization

To ensure the robustness and accuracy of results, the SNPs were quality checked to obtain compliant IVs. Principles of SNPs selection were as follows: (A) the SNPs should be strongly associated with exposures; (B) the SNPs should not be related to confounders; (C) the SNPs should be associated with outcomes mediated by the exposures (Burgess et al., 2019). Since the number of eligible IVs (genome-wide statistical significance threshold, *p* < 5 × 10^−8^) was extremely small, the locus-wide significance threshold (*p* < 1 × 10^−5^) was selected to obtain a more comprehensive result (Jia et al., 2019; Lv et al., 2021). Then, to eliminate linkage disequilibrium (LD), a clumping method with *r*^2^ = 0.001 and kb = 10,000 was applied. Lastly, the *F* statistics were calculated to assess the strength of the selected SNPs using the formula:


$$F=\frac{R^{2}\left(N–k–1\right)}{\left[\left(1–R^{2}\right)\;k\right]}$$

In this formula, *R^2^* is the fraction of variability explained by each SNP, *N* is the GWAS sample size, and k is the number of SNPs. A *F* statistic of 10 indicates that there is no convincing evidence of instrument bias (Yengo et al., 2018).

### 2.6. Multivariate MR analysis

Obesity has recently been identified as a major confounder in the association of intestinal diseases, as it is somehow associated with the health outcome under study, while possibly affecting the composition of the microbiome at the same time (Vujkovic-Cvijin et al., 2020). To address this issue and avoid potential bias associated with sample overlap (Burgess et al., 2016), we performed multivariate MR (MVMR) as a sensitivity analysis to correct for measured confounder and the body mass index [BMI, (SD, ~4.8 kg/m^2^)] was employed as the potential confounder. We selected GWAS meta-analyses for BMI that is currently publicly available and has a relatively large sample size (Locke et al., 2015).

### 2.7. Statistical analysis

The inverse variance weighted (IVW) method was used as the primary analysis for MR. The MR-Egger, weighted median, weighted mode and simple mode were utilized as sensitivity analysis methods to assess the robustness of significant results. Outlying genetic variables may have a considerable influence on MR-Egger, leading to inaccurate calculations. Even if all of the IVs are invalid, the MR-Egger method can still produce unbiased estimates. If SNPs providing 50% of the weight are reliable instruments, the weighted median estimate, as the weighted median of the SNP-specific estimates, yields valid results (Bowden et al., 2016). If the most common horizontal pleiotropy value is zero, regardless of the type of horizontal pleiotropy, the simple mode-based estimate is consistent. When the majority of IVs have identical causal estimates, the weighted mode method is still viable even if the remaining instrumental variables do not match the MR method’s conditions for causal inference. For MVMR analysis, the inverse-variance weighted method was employed.

The possible pleiotropic effects were assessed using MR-Egger regression, which provides a valid test of horizontal pleiotropy as well as a valid test of the causal null hypothesis under the instrument strength independent of direct effect assumption (InSIDE) (Bowden et al., 2015). Besides, MR pleiotropy residual sum and outlier (MR-PRESSO) test also was performed to identify possible horizontal pleiotropy and eliminate pleiotropy impacts by removing outliers (Verbanck et al., 2018). Furthermore, Cochran’s Q-statistic was used to detect the heterogeneity. Odds ratios (ORs) with 95% confidence intervals (CIs) were used to represent the relationship between gut microbiota/gut microbial metabolites and LBP. A reverse causality analysis is also performed to evaluate the reverse causal relation-ship.

A value of *p* of <0.05 was considered as the significance threshold. To adjust for multiple testing (multiple exposures), the statistical significance of the MR effect estimates was defined at a Benjamini–Hochberg false discovery rate (FDR) of less than 5%. All the analyses were conducted by applying packages “TwoSampleMR,” “MRPRESSO” and “MendelianRandomization” in R version 4.2.1. The analysis codes were showed in Supplementary Table 1.

## 3. Results

### 3.1. Selection of instrumental variables

Initially, 13,749 (gut microbiota; locus-wide significance level, *p* < 1 × 10^−5^) and 66 (gut microbial metabolites; locus-wide significance level, *p* < 1 × 10^−5^) SNPs were identified as potential IVs from large-scale GWAS after removing palindromic SNPs (Supplementary Tables 2, 3). It contained 211 bacterial traits, which included five biological classifications: phylum (245 SNPs), class (425 SNPs), order (523 SNPs), family (803 SNPs), and genus (2,703 SNPs). 8 gut microbial metabolites were identified, including BHB (5 SNPs), betaine (13 SNPs), carnitine (12 SNPs), choline (7 SNPs), GABA (11 SNPs), propionic acid (3 SNPs), serotonin (8 SNPs) and TMAO (8 SNPs). After clumping and harmonization, 5,078 (*p* < 1 × 10^−5^) and 66 (*p* < 1 × 10^−5^) SNPs were selected as IVs. The *F*-statistics of IVs were all generally greater than 10, indicating no evidence of weak instrument bias. The key features of SNPs, including effect allele, other allele, beta, SE, and value of *p*, were systematically gathered for further analysis (Supplementary Tables 4, 5).

### 3.2. Causal effects of gut microbiota on low back pain

A total of 20 causal associations from gut microbiota features (1 phylum, 2 class, 5 family, 11 genera and 1 order) to LBP traits were identified by the IVW method (Supplementary Tables 6, 7).

**Figure 2:**
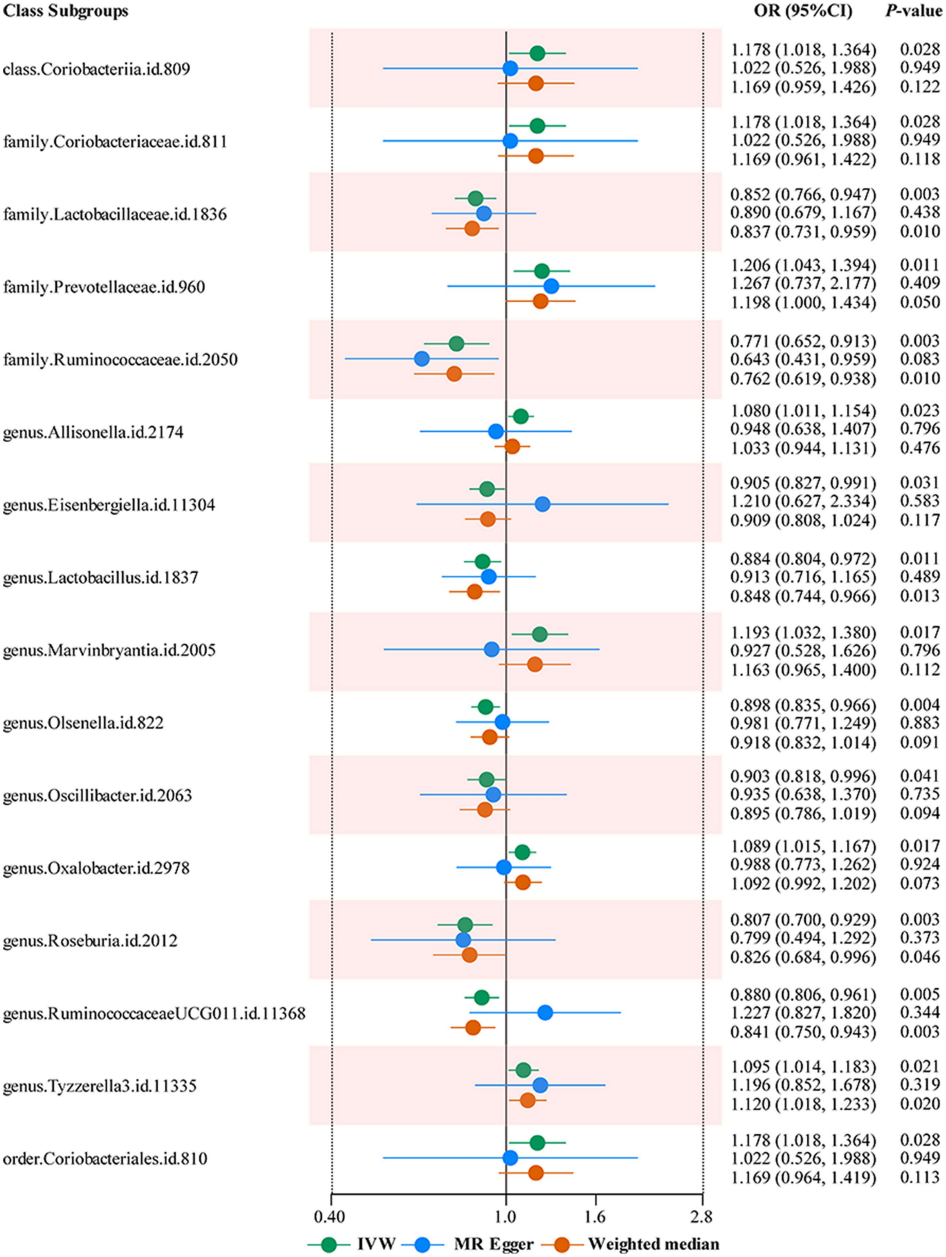
Forest plots summarizing the Mendelian randomization results of gut microbiota taxa with a causal relationship to low back pain. OR, odds ratio; CI, confidence interval; IVW, inverse variance weighted; MR, Mendelian randomization.

The results of IVW analyses demonstrated that genetically greater abundance of class *Coriobacteriia* (OR: 1.178, 95% CI: 1.018–1.364*, p* = 0.028), family *Coriobacteriaceae* (OR: 1.178, 95% CI: 1.018–1.364, *p* = 0.028), family *Prevotellaceae* (OR: 1.206, 95% CI: 1.043–1.394, *p* = 0.011), genus *Allisonella* (OR: 1.080, 95% CI: 1.011–1.154, *p* = 0.023), genus *Marvinbryantia* (OR: 1.193, 95% CI: 1.032–1.380, *p* = 0.017), genus *Oxalobacter* (OR: 1.089, 95% CI: 1.015–1.167, *p* = 0.017), genus *Tyzzerella3* (OR: 1.095, 95% CI: 1.014–1.183, *p* = 0.021), and order *Coriobacteriales* (OR: 1.178, 95% CI: 1.018, *p* = 0.028) were positively correlated with the risk of LBP (only low back pain). Besides, the genetically predicted abundance of class *Clostridi* (OR: 0.88, 95% CI: 0.785–0.986, *p* = 0.028), family *Lactobacillaceae* (OR: 0.852; 95% CI: 0.766–0.947, *p* = 0.003), family *Ruminococcaceae* (OR: 0.771, 95% CI: 0.652–0.913, *p* = 0.003), family *Rikenellaceaegenus* (OR: 0.894, 95% CI: 0.804–0.994, *p* = 0.039), genus Turicibacte (OR: 0.907, 95% CI: 0.835–0.985, *p* = 0.021), genus *Eisenbergiella* (OR: 0.905, 95% CI: 0.827–0.991, *p* = 0.031), genus *Lactobacillus* (OR: 0.884, 95% CI: 0.804–0.972, *p* = 0.011), genus *Olsenella* (OR: 0.898, 95% CI: 0.835–0.966, *p* = 0.004), genus *Oscillibacter* (OR: 0.903, 95% CI: 0.818–0.996, *p* = 0.041), genus *Roseburia* (OR: 0.807, 95% CI: 0.700–0.929, *p* = 0.003) and genus *RuminococcaceaeUCG011* (OR: 0.880, 95% CI: 0.806–0.961, *p* = 0.005) were correlated with a reduced risk of LBP (only low back pain) (Figure 2).

**Figure 3:**
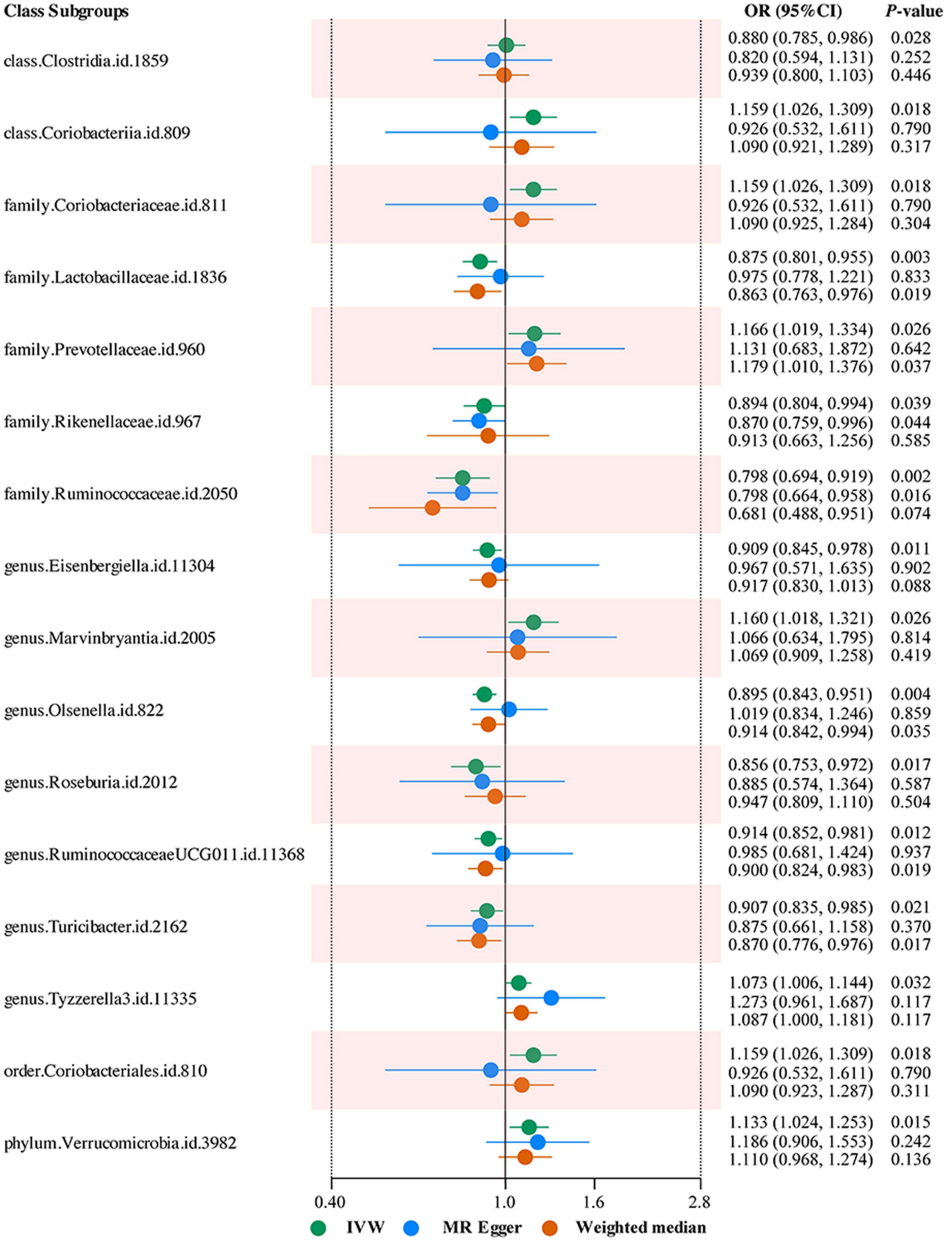
Forest plots summarizing the Mendelian randomization results of gut microbiota taxa with a causal relationship to low back pain or/and sciatica.

Moreover, the IVW results demonstrated that class *Coriobacteriia* (OR: 1.159, 95% CI: 1.026–1.309, *p* = 0.018), family *Coriobacteriaceae* (OR: 1.159, 95% CI: 1.026–1.309, *p* = 0.018), family *Prevotellaceae* (OR: 1.166, 95% CI: 1.019–1.334, *p* = 0.026), genus *Marvinbryantia* (OR: 1.160, 95% CI: 1.018–1.321, *p* = 0.026), genus *Tyzzerella3* (OR: 1.073, 95% CI: 1.006–1.144, *p* = 0.032), order *Coriobacteriales* (OR: 1.159, 95% CI: 1.026–1.309, *p* = 0.018), phylum *Verrucomicrobia* (OR: 1.133, 95% CI: 1.024–1.253, *p* = 0.015) were positively correlated with the risk of LBP or/and sciatica. Moreover, class *Clostridia* (OR: 0.880, 95% CI: 0.785–0.986, *p* = 0.028), family *Lactobacillaceae* (OR: 0.875, 95% CI: 0.801–0.955, *p* = 0.003), family *Rikenellaceae* (OR: 0.894, 95% CI: 0.804–0.994, *p* = 0.039), family *Ruminococcaceae* (OR: 0.798, 95% CI: 0.694–0.919, *p* = 0.002), genus *Eisenbergiella* (OR: 0.909, 95% CI: 0.845–0.978, *p* = 0.011), genus *Olsenella* (OR: 0.895, 95% CI: 0.843–0.951, *p* = 0.004), genus *Roseburia* (OR: 0.856, 95% CI: 0.753–0.972, *p* = 0.017), genus *RuminococcaceaeUCG011* (OR: 0.914, 95% CI: 0.852–0.981, *p* = 0.012), genus *Turicibacter* (OR: 0.907, 95% CI: 0.835–0.985, *p* = 0.021), were negatively correlated with the risk of LBP or/and sciatica (Figure 3).

### 3.3. Causal effects of gut microbial metabolites on low back pain

IVW results indicated that a higher genetically predicted BHB (OR: 1.067, 95% CI: 1.002–1.135, *p* = 0.043) was associated with the higher risk of LBP or/and sciatica. Besides, a higher genetically predicted TMAO were associated with the higher risk of LBP (OR: 1.064, 95% CI: 1.008–1.122, *p* = 0.023). In addition, there was no indication of a causal relationship between the remaining six gut microbial metabolites and LBP (Supplementary Tables 8, 9).

### 3.4. Benjamini–Hochberg corrected test, sensitivity analysis and reverse analysis

Results from the Benjamini–Hochberg Corrected test revealed that a lower level of family *Ruminococcaceae* and family *Lactobacillaceae* retains a strong causal relationship with the higher risk of LBP (IVW FDR-corrected *p* = 0.045) (Supplementary Table 7). Q statistics from IVW test and MR-Egger regression showed no evidence of heterogeneity in most causal relationships (*p* > 0.05) (Supplementary Tables 10, 11). None of the MR-Egger regression intercepts deviated from zero, indicating that there was no indication of horizontal pleiotropy (all intercept *p* > 0.05) (Supplementary Tables 12, 13). MR-PRESSO test uncovered no evidence of horizontal pleiotropy in causal relationships (*p* > 0.05) (Supplementary Table 14). Besides, Leave-one-out analysis indicated that the causal association signals were not driven by any single SNP (Supplementary Tables 15, 16). In reverse MR analysis, there was no evidence of a causal effect of LBP on gut microbiota (Supplementary Table 17).

### 3.5. Exploration of BMI as potential confounding factor

Obesity was recently identified as a major confounding factor in microbiome-disease associations. We perform a multivariable MR to check if the causal effects were still robust by the inclusion of obesity. After adjusted for BMI, the IVW results of MVMR analyses demonstrated that genus *Allisonella* (OR: 1.106, 95% CI: 1.030–1.188, *p* = 0.006), genus *Eisenbergiella* (OR: 0.903, 95% CI: 0.818–0.996, *p* = 0.041), TMAO (OR: 1.064, 95% CI: 1.010–1.121, *p* = 0.019) were significantly correlated with the risk of LBP (only low back pain), and genus *Eisenbergiella* (OR: 0.902, 95% CI: 0.831–0.980, *p* = 0.015), genus *Olsenella* (OR: 0.921, 95% CI: 0.859–0.987, *p* = 0.020) and BHB (OR: 1.051, 95% CI: 1.011–1.093, *p* = 0.012) were significantly correlated with the risk of LBP or/and sciatica. However, the remaining associations found may be confounded to some extent by BMI (Supplementary Table 18).

## 4. Discussion

**Figure 4:**
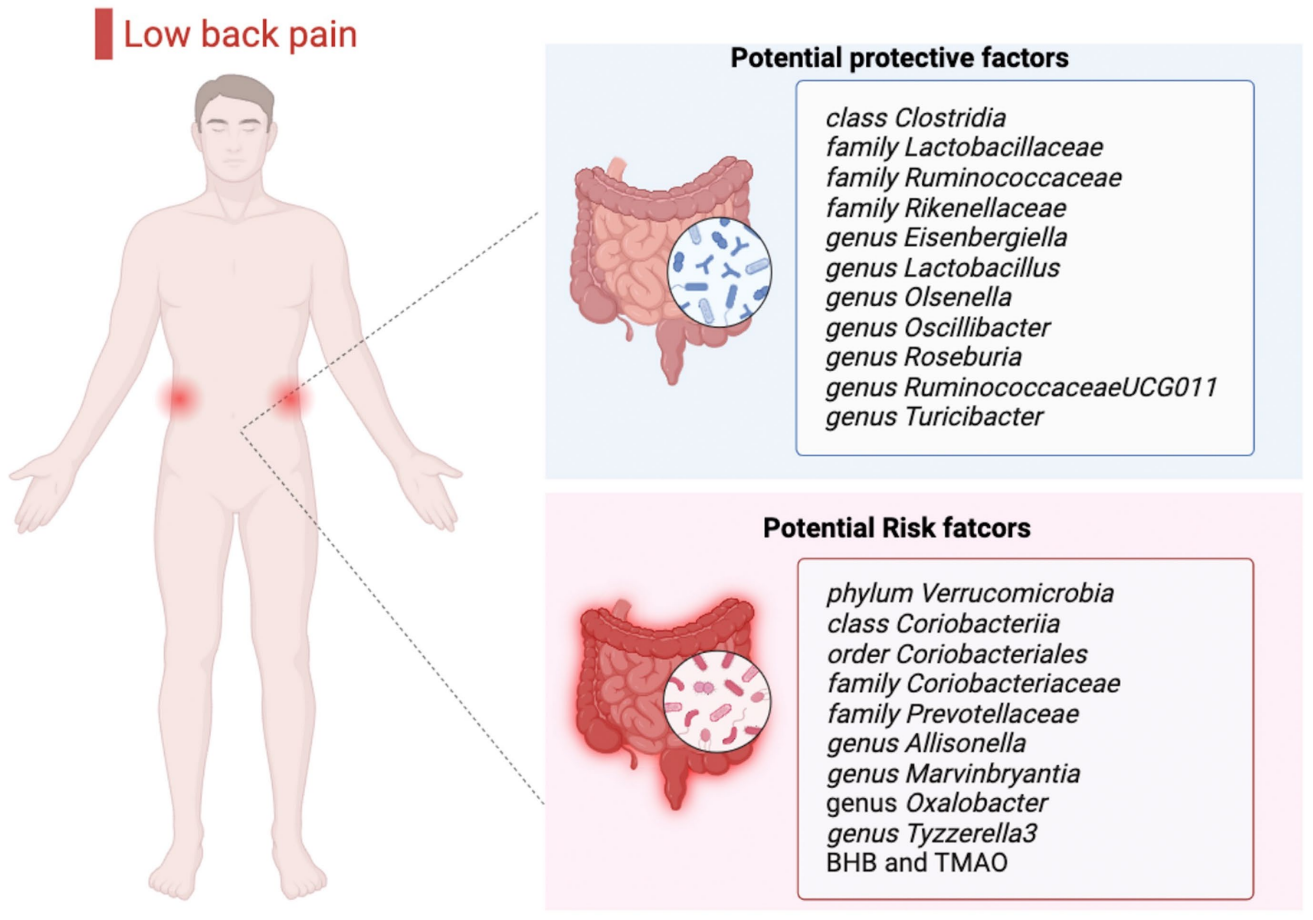
Causal links between gut microbiota, gut microbial metabolites and low back pain. BHB, β-hydroxybutyric acid; TMAO, trimethylamine N-oxide.

To the best of our knowledge, this is the first MR analysis report to establish the causal relationship between gut microbiota/metabolites and LBP. In this two-sample MR study, we identified that 20 gut microbial taxa and 2 gut microbial metabolites were causally correlated with LBP (Figure 4). However, their effects were substantially reduced in MVMR analyses incorporating BMI.

The potential mechanisms of the gut microbiota involving in the pathogenesis of LBP was discussed in many prior studies. It’s believed that all the elements comprising the lumbar spine, such as muscles, fascia, ligaments, tendons, joints, neurovascular elements, vertebrae and intervertebral discs can contribute to LBP, and intervertebral disc degeneration is regarded as one of the most likely causes (Khan et al., 2017; Knezevic et al., 2021). Rajasekaran et al. (2020) found a higher abundance of *Oxalobacter* in India human degenerated intervertebral disc and *Lactobacillus* was found to be abundant in normal disc compared to disc herniation, which follows a similar trend to our results. In lumbar disc herniation mouse models, a relatively high abundance of *Ruminococcaceae* in gut is associated with improved behavior, increased cell proliferation and decreased apoptosis (Wang et al., 2021). Therefore, gut microbiota may affect LBP mediating dysbiosis of microbiota in intervertebral disc. LBP that extends into the leg, usually below the knee, is radicular pain or neuropathic pain (previously called sciatica). The prevalence of neuropathic pain has ranged between 16 and 55% in patients with chronic LBP (Knezevic et al., 2021). Rothhammer et al. discovered that dietary tryptophan metabolized by *Clostrid* could act directly on astrocytes, limiting inflammation and providing neuroprotective effects to reduce neuropathic pain in mice (Rothhammer et al., 2018). Our results agreed with the probiotic effect of *Clostrid* from previous studies. Analysis of 16S rRNA gene sequencing of fecal displayed that *Verrucomicrobia* is highly correlated with neuropathic pain according to animal experiment (Li R. et al., 2022). Furthermore, clinical research found that *Verrucomicrobia* is increased in the gut of patients with neuralgia (Zhang et al., 2019; Lin et al., 2020).

Except for disc generation and radicular pain mentioned above, facet arthropathy, myofascial pain, spondyloarthropathies and sacroiliac joint pain all contribute to the pathogenesis of LBP (Knezevic et al., 2021). Facet joints that connect adjacent vertebrae are also prone to degenerative changes, most commonly osteoarthritis. In osteoarthritis mouse models, *Lactobacillaceae* treatment was founded to decrease pain severity and cartilage destruction (Jhun et al., 2021). Similarly, a community-based observational study in China including 1,388 participants provided the evidence that a low relative abundance of *Roseburia* is associated with symptomatic osteoarthritis (Wei et al., 2021). Additionally, several studies had demonstrated that a decreased relative abundance of the genus *Roseburia* is associated with inflammatory diseases (Tamanai-Shacoori et al., 2017; Quiroga et al., 2020; Nie et al., 2021). The function of reliving inflammatory may be a potential approach of *Roseburia* affecting LBP. Chevalier et al. had confirmed that transplantation of *Turicibacter* in gut could prevent bone loss in female mice, so that *Turicibacter* could serve as a protective factor for osteoporosis (Chevalier et al., 2020). Likewise, muscles can also be pain generators of LBP, A large-scale survey in Japan including 848 participants indicated that *Eisenbergiella* is positively associated with skeletal muscle mass/body weight, which might help increase the resistibility of LBP (Sugimura et al., 2022). In line with these studies, our study suggested that the increased relative abundance of *Lactobacillus, Rikenella, Lactobacillaceae, Roseburia, Ruminococcaceae, Turicibacter, Eisenbergiella* was causally associated with a lower risk of LBP.

As a family of chronic and inflammatory autoimmune disease, spondyloarthropathy affect multiple joints, with ankylosing spondylitis preferentially affecting the low back and sacroiliac joint (Shen et al., 2022). Analysis of 16S rRNA gene sequencing of fecal in 85 patients and 62 healthy controls in China displayed that *Prevotella* is highly correlated with ankylosing spondylitis (Zhou et al., 2020), which is consistent with our results. In addition, the prevalence for spondyloarthropathies was 0.05–0.25% for enteropathic axial arthritis and *Olsenella* was found to be associated with decreased disease activity index in inflammatory bowel disease mouse models (Reveille, 2011; Zhang et al., 2020). At the same time, extensive research have detected a relationship between vitamin D and LBP (Zadro et al., 2017; Al-Taiar et al., 2020; Kanaujia et al., 2021; Xu et al., 2021). A large-scale randomized controlled trial demonstrated that vitamin D could reduce the risk of autoimmune diseases including inflammatory arthropathy (Hahn et al., 2022). The mechanism that provides rationale for the link between vitamin D and the risk of LBP remains ambiguous. One the one hand, vitamin D is recognized to induce changes in bone metabolism and could regulate the inflammatory cytokines that control pain (Xu et al., 2021; Murdaca et al., 2022). On the other hand, vitamin D deficiency deeply influences the microbiome by altering the microbiome composition and the integrity of the gut epithelial barrier (Murdaca et al., 2021a). Multiple studies have shown that vitamin D deficiency is associated with microbiome dysbiosis, with consequent increases in inflammatory disorders (Murdaca et al., 2019, 2021b). Therefore, there may be a link between gut microbiota, vitamin D and LBP.

A two-sample bidirectional MR study provided robust evidence that *Allisonella* may be a risk of multisite chronic pain (Lin et al., 2022), including LBP, which supported our results. *Coriobacteriales* is an order of *Coriobacteriia*, whose subordinate family comprises *Coriobacteriaceae.* Available literature indicated that *Coriobacteriia* is significantly more abundant in bipolar disorder and colorectal carcinomas (Painold et al., 2019), whereas there is little evidence about the relationship with pain. The exact mechanism of these gut microbial taxa on the development of LBP warrants additional investigations.

On the other hand, gut microbial metabolites, as the main way for gut microbiota to affect host function, are involved in the occurrence and development of various diseases (Bai et al., 2022; Teunis et al., 2022; Yu et al., 2022). In recent years, the functional mechanism of gut microbial metabolites in nervous-related disorders have received extensive attention. Among them, gut microbial metabolites play a regulatory role in the development of a variety of chronic pain, such as visceral pain, inflammatory pain, neuropathic pain and headache (Li J. S. et al., 2022). TMAO is a gut microbiota-derived metabolite produced from choline and carnitine, which are essential nutrients contained in many foods, including red meat, eggs and dairy (Koeth et al., 2013; Hazen and Brown, 2014). TMAO is involved in pain generation and transmission by significantly triggering oxidative stress and decrease anti-inflammatory factor (Silvestre et al., 2020; Ko et al., 2022). The result of our study was in accordance with the most available evidence. As one of the endogenous ketone metabolites, BHB is a small, water-soluble and lipid-derived molecule (Nosadini et al., 1989; Fukao et al., 2004; Cheng et al., 2019). Previous research indicated that an increase levels of BHB in plasma is associated with reduced pain sensitivity (Lautenbacher et al., 1991; Smith et al., 2013) and caloric restriction could alleviate complete Freund’s adjuvant-induced inflammatory pain *via* elevating BHB expression (Liu et al., 2022). Interestingly, after treatment with non-steroidal anti-inflammatory drugs, early postpartum Holstein Friesian dairy cows experienced pain relief and a reduction in serum BHB concentrations (Schmitt et al., 2023). Here, we observed elevated BHB was a risk factor of LBP. The exact mechanism of BHB on the pathogenesis of LBP warrants verification.

The advantages of this study as follows: MR employs genetic variants as environmental exposure proxies to identify the causal relationship between an exposure and a disease outcome. Because genetic differences are assumed to be assigned at random before birth, they are highly independent of environmental variables and established well before sickness onset, avoiding residual confounding and reverse causation problems that limit traditional observational studies (Smith and Ebrahim, 2003). Then, this study takes advantage of publicly available datasets to gain more precise estimates and greater statistical power due to the large sample sizes of GWAS. Last but not least, MVMR as a sensitivity analysis to correct for measured confounder and the BMI is believed to increase the robustness and reproducibility in resolving the gut microbiome that are truly associated with LBP. In brief, this study was adequately powered to detect a significant association between gut microbiota/gut microbial metabolites and LBP.

However, there are some limitations in this study. First, to limit population stratification bias, the majority of participants in the GWAS pooled data included in our study were of European ancestry, which may partially bias our estimates. Though some previous studies using 16S rRNA-based phylogeny have also demonstrated that LBP is potentially related to gut dysbacteria (such as *Oxalobacter, Lactobacillus, Prevotella, Roseburia and Eisenbergiella* etc.) among Asian population, causal relationship between gut microbiota/metabolites and LBP in people from other regions remain unclear (Rajasekaran et al., 2020; Zhou et al., 2020; Wei et al., 2021; Sugimura et al., 2022). Second, due to a lack of demographic data (e. g. gender and ethnicity) in the original study, additional subgroup analysis was not feasible. Third, science the SNPs obtained using the genome-wide statistical significance threshold (*p* < 5 × 10^−8^) were insufficient for further analysis, only the SNPs that met the locus-wide significance level (*p* < 1 × 10^−5^) were identified. These limits reduced the results’ generalizability and may have weakened the study’s accuracy. After a causal relationship is demonstrated, the next step is exploring possible mechanisms that allow the microbiome to affect the host health.

In conclusion, we comprehensively confirmed the causal association between gut microbiota/gut microbial metabolites and LBP. Nine bacterial features and two gut microbial metabolites showed a positive causal direction with LBP, whereas another eleven bacterial features showed a negative causal direction with LBP. These strains may become novel biomarkers and provide insights for the treatment and prevention of LBP.

## Data availability statement

The datasets presented in this study can be found in online repositories. The names of the repository/repositories and accession number(s) can be found in the article/Supplementary material.

## Author contributions

MS, YT, and TZ designed the study and wrote the manuscript. MS, YT, WK, and SZ analyzed data and performed the literature search. YT and TZ supervised the manuscript. MS and YT contributed equally to this work. All authors were involved in writing the manuscript, contributed to the article, and approved the submitted version.

## Funding

This work was supported by the National Key R&D Program of China (No. 2018YFC2001800 to TZ), the National Natural Science Foundation of China (No. 81671062 to TZ), Post-Doctor Research Project, West China Hospital, Sichuan University (No. 2021HXBH059 to YT), the Natural Science Foundation of Sichuan Province (No. 2023NSFSC1780 to YT).

## Conflict of interest

The authors declare that the research was conducted in the absence of any commercial or financial relationships that could be construed as a potential conflict of interest.

## Publisher’s note

All claims expressed in this article are solely those of the authors and do not necessarily represent those of their affiliated organizations, or those of the publisher, the editors and the reviewers. Any product that may be evaluated in this article, or claim that may be made by its manufacturer, is not guaranteed or endorsed by the publisher.

## Footnotes

^1^https://r5.finngen.fi/
